# Modeling spatial determinates of teenage pregnancy in Ethiopia; geographically weighted regression

**DOI:** 10.1186/s12905-021-01400-7

**Published:** 2021-06-24

**Authors:** Seblewongel Tigabu, Alemneh Mekuriaw Liyew, Bisrat Misganaw Geremew

**Affiliations:** 1 Oromyaa Regional Health Bureau, Addis Ababa, Ethiopia; 2 Department of Epidemiology and Biostatistics, Institute of Public Health, College of Medicine and Health Sciences and Comprehensive Specialized Hospital, University of Gondar, P.O. Box 196, Gondar, Ethiopia

**Keywords:** Teenage pregnancy, Spatial determinates, Geographic weighted regression, Ethiopia

## Abstract

**Background:**

In developing countries, 20,000 under 18 children give birth every day. In Ethiopia, teenage pregnancy is high with Afar and Somalia regions having the largest share. Even though teenage pregnancy has bad maternal and child health consequences, to date there is limited evidence on its spatial distribution and driving factors. Therefore, this study is aimed to assess the spatial distribution and spatial determinates of teenage pregnancy in Ethiopia.

**Methods:**

A secondary data analysis was conducted using 2016 EDHS data. A total weighted sample of 3381 teenagers was included. The spatial clustering of teenage pregnancy was priorly explored by using hotspot analysis and spatial scanning statistics to indicate geographical risk areas of teenage pregnancy. Besides spatial modeling was conducted by applying Ordinary least squares regression and geographically weighted regression to determine factors explaining the geographic variation of teenage pregnancy.

**Result:**

Based on the findings of exploratory analysis the high-risk areas of teenage pregnancy were observed in the Somali, Afar, Oromia, and Hareri regions. Women with primary education, being in the household with a poorer wealth quintile using none of the contraceptive methods and using traditional contraceptive methods were significant spatial determinates of the spatial variation of teenage pregnancy in Ethiopia.

**Conclusion:**

geographic areas where a high proportion of women didn’t use any type of contraceptive methods, use traditional contraceptive methods, and from households with poor wealth quintile had increased risk of teenage pregnancy. Whereas, those areas with a higher proportion of women with secondary education had a decreased risk of teenage pregnancy. The detailed maps of hotspots of teenage pregnancy and its predictors had supreme importance to policymakers for the design and implementation of adolescent targeted programs.

## Background

Annually 16 million births were from teenagers which is 11% of total birth [1] Though the global adolescent birth rate has declined from 65 births per 1000 to 45 births per 1000 from 1990 to 2015 [2], in developing countries still about 20,000 under 18 children give birth every day [1]. One-third of the Ethiopian population is young people. Though the Ethiopian government has planned to reduce teenage pregnancy to 3% by 2020 [3], still 13% of teenagers have started childbearing in Ethiopia with the highest prevalence in Afar and Somalia regions [4].

For the past 100 years, the average age of menarche has become 4 months earlier every 10 years which leads to the early initiation of sexual activity. In relation to poor contraceptive use, it has led to unwanted pregnancy and childbirth [5]. Teenage delivery is related to several adverse pregnancy outcomes such as low Apgar score, preterm babies, low birth weight, neonatal mortality, and stillbirth [6–8]. Besides, maternal complications like maternal death [9] perineal tear, anemia. Preeclampsia, eclampsia, preterm labor, postpartum hemorrhage, hypertensive disorders of pregnancy, premature rupture of membrane, cephalo pelvic disproportion, malpresentation, abortion, and delivery by caesarian section are also common mother related bad consequences of teenage pregnancy [8, 10, 11].

Moreover, teenage pregnancy has series long term problems that affect the girls themselves and their community. It leads them to less educational attainment and high school dropout, disease exposure and economic problems. This will not end by themselves rather their siblings are also prone to these problems and are more likely to give birth as teenagers, face unemployment and engage in criminal acts at some time during their adolescence [10, 12]

In Ethiopia, the prevalence of teenage pregnancy is different in different regions which range from 7.70% in Arbaminch town south Ethiopia [12], 20.40% in Assosa [13] to 28.6% in northeast Ethiopia [14]. Besides, an education level [15, 16], age, Employment status [15], occupation [13], wealth status [17, 18], media exposure, marital status [19], contraceptive use [13, 18] place of residence [20] community poverty, female community unemployment, and community contraceptive us [15] were found to have a significant association with teenage pregnancy.

Focusing on the geographic variation of teenage pregnancy and its driving factors through spatial analysis has valuable evidence for policymakers to reduce teenage pregnancy in Ethiopia [21]. But, none of the previous studies has detected the spatial variation and its determinates in Ethiopia. The core assumption of spatial analysis goes to Tobler’s first law which states that “everything is related to everything else, but near things are more related than distant things’’ [22]. This concept is considered as the core of spatial autocorrelation statistics and is central to every spatial analytical technique including the analytical conceptions of geographic space. Therefore, the current study aimed to answer the following questions through a spatial analytic approach. First, where the hotspot (most risk areas) areas of teenage pregnancy are concentrated (clustered) in Ethiopia? Second, what are the driving factors for such spatial variations of teenage pregnancy in Ethiopia? Thus, this study is aimed at exploring the spatial variation and modeling the spatial determinates of teenage pregnancy in Ethiopia.

## Methods

### Data source, study design, and setting

This study utilized secondary data from 2016 Ethiopian Demographic and Health Survey (EDHS). The survey data were downloaded from the Measure DHS website after reasonable request and data use permission was fully guaranteed. The 2016 EDHS is part of the worldwide MEASURE DHS project which was funded by the United States Agency for International Development (USAID) and was implemented by the Ethiopian Central Statistical Agency. A DHS is undertaken every 5 years and the 2016 survey is the fourth Demographic and Health Survey in Ethiopia which covers all the nine regions and two administrative cities.

### Sample size and sampling procedure

The Ethiopian Demographic and Health Survey program (EDHS) has collected data on nationally representative samples of all age groups and key indicators. The information on the sociodemographic, socioeconomic, and maternal-related variables was included in the survey. A stratified two-stage cluster sampling procedure was employed to select study participants. In the 2016 survey, a total of 645 EAs (202 urban and 443 rural) were selected. From these enumeration areas, 18,008 households and from those households a total of 15,683 reproductive-age women were included in the survey. The relevant information on the sampling procedure and data quality can be accessed elsewhere [4]. For the current study, a total of 3381(weighted sample) teenagers (15–19 years old) were included.

### Study variables

#### Dependent variable

Teenage pregnancy: It is a composite binary outcome variable that refers to the pregnancy experience of a woman between the ages of 15–19 years. History of birth before age of 19 or being pregnant at the time of the interview was considered as teenage pregnancy. Therefore, it was categorized in such a way that 0 = no pregnancy before age 19 and 1 = pregnancy experienced before the age of 19 years. Finally, the weighted proportion of teenage pregnancy per cluster which is a continuous variable was used for spatial analysis including spatial regression analysis.

#### Independent variables

The aggregated community variables such as community poverty (the proportion of the two lowest wealth quintiles), community contraceptive use (the proportion of women who didn’t use any type of contraceptive), community traditional contraceptive use (the proportion of women who use traditional contraceptive methods), community women education (proportion of women with no education), female community employment (the proportion of unemployed women), community media exposure (the proportion of women who were not exposed to television, radio or reading newspaper) community health insurance coverage (proportion of women who were not covered by health insurance) and community illiteracy (the proportion of women unable to read and write) were considered as candidate independent variables for the spatial regression models.

### Data management and analysis

Descriptive analyses were performed using Stata version 14 statistical software. Whereas the spatial analysis was performed using ArcGIS 10.7. Before conducting spatial analysis, the weighted proportions of teenage pregnancy (outcome variable) and candidate predictor variables performed in stata and were exported to ArcGIS. A detailed explanation of the weighting procedure can be found elsewhere [23].

### Spatial analysis

#### Spatial autocorrelation

Spatial autocorrelation rises from the concept of correlation or dependency. Geographically close areas are more related than distant areas. In global autocorrelation the concept is stationary. The correlation between nearby or connected observations will remain the same. Moran I is an indicator of spatial autocorrelation in the range of − 1 to 1. The value being positive shows that close areas have similar values whereas a negative value is an indicator if dissimilarity between adjacent values [24]. The global moran’s I was computed as follows [25]

$${\text{I}} = \frac{{{\text{n}}\sum\nolimits_{{\text{i}}}^{{\text{n}}} {\sum\nolimits_{{\text{j}}}^{{\text{n}}} {{\text{wij}}} } \left( {{\text{yi}} - \overline{{\text{y}}} } \right)\left( {{\text{yj}} - \overline{{\text{y}}} } \right)}}{{\left( {\sum\nolimits_{{\text{i}}}^{{\text{n}}} {\sum\nolimits_{{\text{j}}}^{{\text{n}}} {{\text{wij}}} } } \right)\sum\nolimits_{{\text{i}}} {\left( {yi - \bar{y}} \right)^{2} } }}$$

where yi represents the vector of observations at n different locations, and wij are elements of a spatial weight matrix.

#### Hot spot analysis

Hot spot analysis identifies statistically significant clustering areas using vectors calculates The Getis–Ord Gi statistic the resultant Z score and *p* value will identify where the high or low values cluster spatially. The hot spot area is where high values of the given data are surrounded by similar high values to the opposite where low values are surrounded by similar low values give the cold spot areas [26].

#### Spatial scan statistics

Satscan analyzes spatial–temporal and space–time data using spatial–temporal or space–time scan statistics. It is used to perform geographical surveillance of disease and to detect areas of significantly high or low rates. In the Bernoulli-based model pregnant teenagers were taken as cases and non-pregnant teenagers as controls to determine the geographical locations of statistically significant clusters of teenage pregnancy using kuldorff sat scan version 9.6 software The default maximum spatial cluster size of < 50% of the population was used. The primary and secondary clusters were detected and ranked according to the likelihood ratio test, based on 999 Monte Carlo replications [27]

### Spatial regression analysis

#### Ordinary least squares (OLS) regression

After detecting the hot spot areas of teenage pregnancy, spatial regression modeling was performed to identify predictors of the observed spatial clustering of teenage pregnancy. So first ordinary least square regression was conducted. Findings from the ordinary least squares (OLS) regression are only reliable if the regression model satisfies all of the assumptions that are required by this method. The coefficients of explanatory variables in a properly specified OLS model should be statistically significant and have either a positive or negative sign. Besides, there should not be a correlation among explanatory variables (free from multicollinearity). The model should be unbiased (heteroscedasticity or non-stationarity). The residuals should be normally distributed and revealed no spatial patterns. The model should include key explanatory variables. The residuals must be free from spatial autocorrelation [28]. Thus, these assumptions were checked accordingly. The OLS regression equation [29] is given as:

$$Y_{i} = \beta + \mathop \sum \limits_{{K = 1}}^{P} \left( {\beta _{k} X_{{ik}} ~} \right) + \in _{i} ~$$

where i = 1, 2,…n; β0, β1, β2, …βp are the model parameters, yi is the outcome variable for observation i, *X*_*ik*_ are explanatory variables and  _1_, ∈_2_, … ∈_*n*_ are the error term/residuals with zero mean and homogenous variance σ2**.**

To identify a model that fulfills the assumption of the OLS method, exploratory regression identifies models with high Adjusted R2 values. Besides, it identifies models that meet all of the assumptions of the OLS method [30].

#### Geographically weighted regression (GWR)

A variable that is a strong predictor in one cluster may not necessarily be a strong predictor in another cluster. This type of cluster variation (non-stationary) can be identified through the use of GWR. In this context, GWR can help to answer the question: “Does the association vary across space?” Unlike OLS that fits a single linear regression equation to all of the data in the study area, GWR creates an equation for each DHS cluster. While the equation in OLS is calibrated using data from all features (cluster in this case), GWR uses data from nearby features. Thus, the GWR coefficient takes different values for each cluster [31] Maps of the coefficients associated with each explanatory variable, which are produced using the GWR, provide guidelines for targeted interventions. The GWR model [32]can be written as:

$$~Y_{{i~}} = \beta _{{O~}} (u_{{i~}} v_{{i~}} ) + \mathop \sum \limits_{{k = 1}}^{p} \beta _{{k~}} \left( {u_{{i~}} v_{{i~}} } \right)X_{{ik~ + ~ \in _{i} }}$$

where yi are observations of response y, *u*_*i*_*v*_*i*_ are geographical points (longitude, latitude), $\beta _{{k~}} (u_{{i}} v_{{i}} )$ (k = 0, 1 … p) are p unknown functions of geographic locations *u*_*i*_*v*_*i*_, *X*_*ik*_ are explanatory variables at location *u*_*i*_*v*_*i*_, i = 1, 2, … n and $\in _{i}$ are error terms/residuals with zero mean and homogenous variance (σ2).

## Results

Table 1 presents the weighted proportion of teenage pregnancy by region. The overall prevalence of teenage pregnancy was 12.80% (95%CI 11.73%, 13.95%) in the current study. Of all the nine regions and two administrative cities Afar region had the highest prevalence of teenage pregnancy.

**Table 1:** the weighted proportion of teenage pregnancy by region

Region	Teenage pregnancy		
	No (%)	Yes	Total (%)
Tigray	243 (88.04)	33 (11.96)	276 (8.16%)
Afar	23 (76.66)	7 (23.34)	30 (0.89%)
Amhara	703 (91.65)	64 (8.35)	767 (22.6)
Oromia	1025 (83.06)	209 (16.94)	1234 (36.4)
Somali	85 (80.95)	20 (19.05)	105 (3.1)
Benishangul Gumuz	29 (85.29)	5 (14.71)	34 (1.0)
SNNP	608 (89.28)	73 (10.72)	681 (20.1)
Gambela	8 (88.88)	1 (11.12)	9 (0.2)
Harari	7 (87.5)	1 (12.5)	8 (0.2)
Addis adaba	211 (97.23)	6 (2.73)	217 (6.4)
Dire dawa	18 (90)	2 (10)	20 (0.5)

*SNNP* South nation nationality and peoples region

### Spatial autocorrelation of teenage pregnancy in Ethiopia

**Fig. 1:**
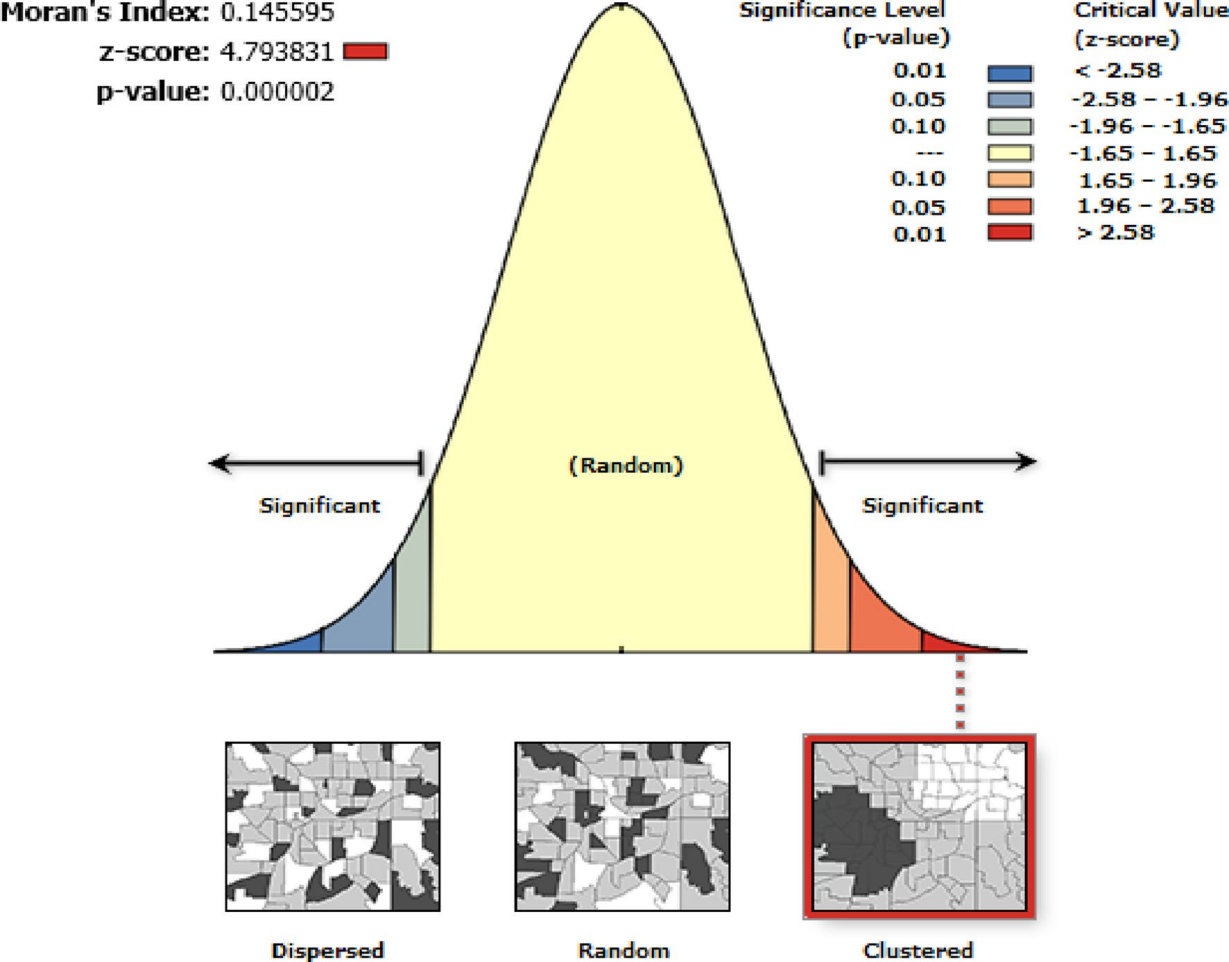
Spatial autocorrelation of teenage pregnancy in Ethiopia

Teenage pregnancy was spatially clustered in Ethiopia with Global Moran’s I = 0.45 and *p* < 0.001 (Fig. 1). The clustered patterns (on the right sides) show high rates of low birth weight occurred over the study area. The Z-score of 4.79 indicated that there is less than 1% likelihood that this clustered pattern could be the result of random chance.

### The hot spot (Getis-Ord Gi*) analysis of teenage pregnancy

**Fig. 2:**
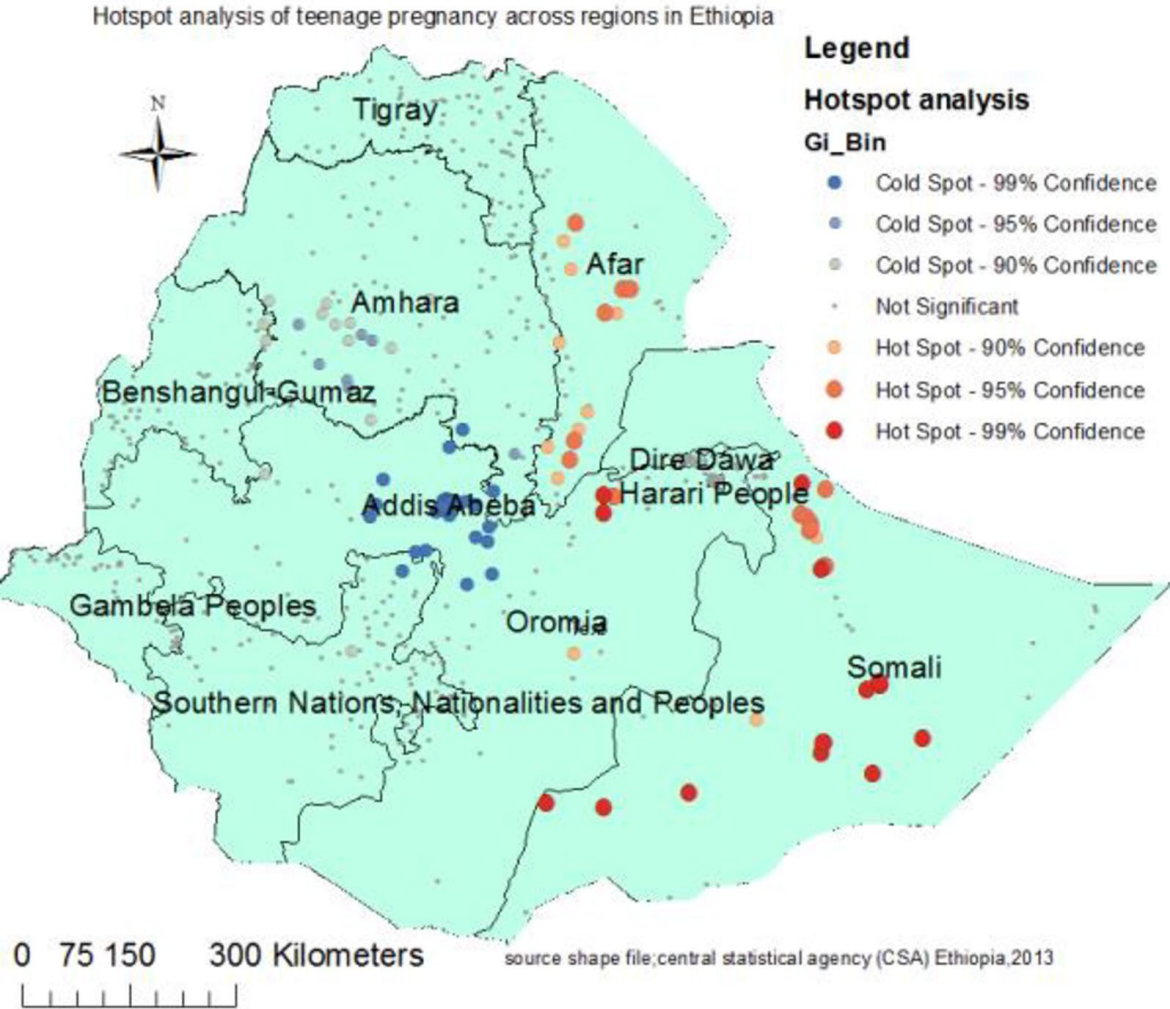
Hot spot analysis of teenage pregnancy across regions in Ethiopia (source; shapefile from central statistical agency, Ethiopia, 2013)

Figure 2 presented the hot spot areas of teenage pregnancy. Accordingly, the central and southern parts of Afar, Hareri peoples region, northern and southwestern part of Somalia regions were at higher risk of teenage pregnancy. Whereas, cold spot (low risk) areas were detected in Addis Ababa, Amhara, Tigray, SNNP, Benshangul gumuz, and Gambela regions (Fig. 2).

### Spatial scan statistics

**Fig. 3:**
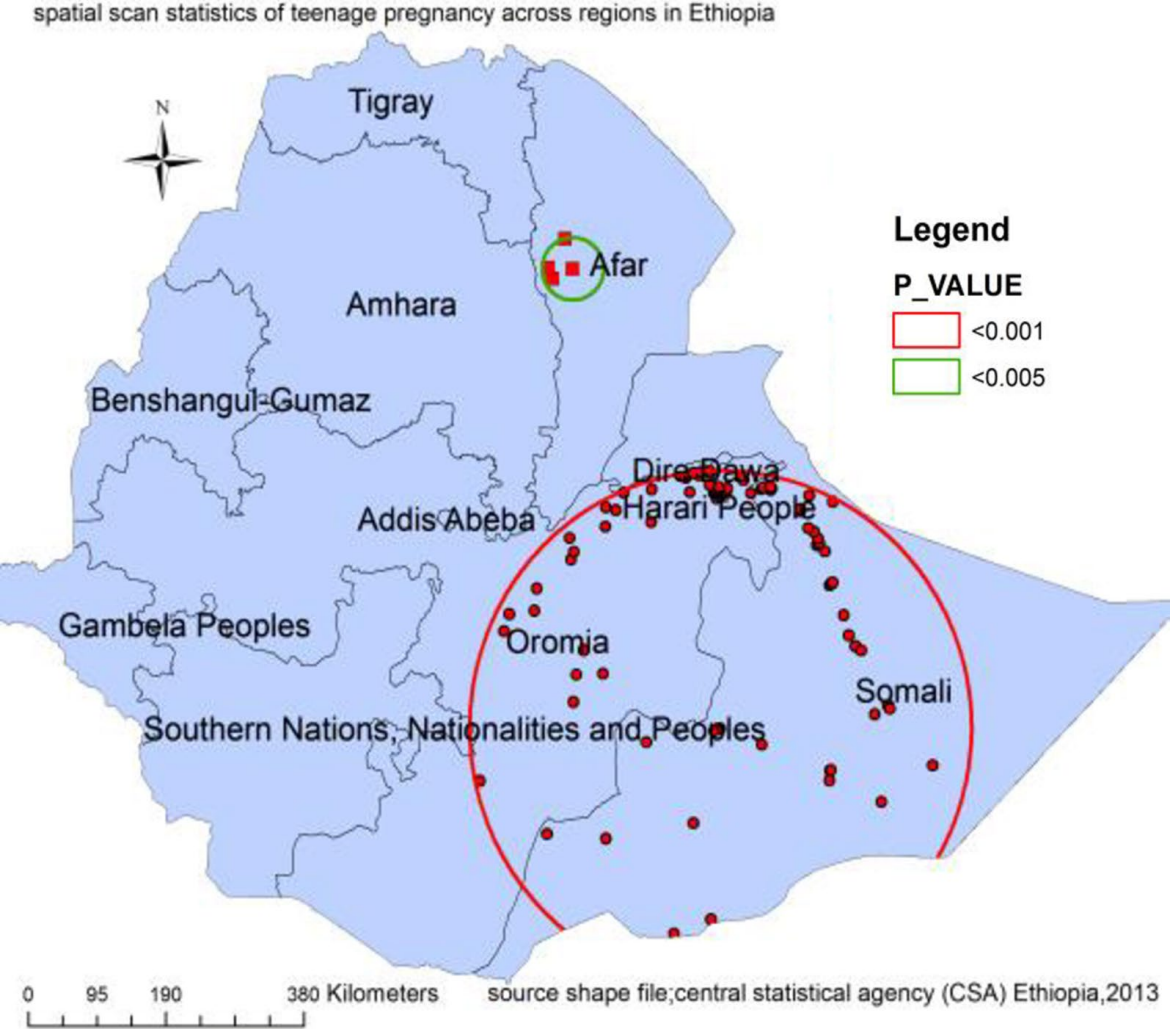
Spatial scan statistics of teenage pregnancy in Ethiopia (source; shapefile from central statistical agency, Ethiopia, 2013)

Spatial scan statistics of teenage pregnancy has two significant scanning windows the first one is located at 6.467859N, 42.175356E which covers most parts of Somali and eastern part of Oromia regions containing 117 clusters with 344.11 km radius, LLR 23.25, *p* value < 0.0001. Teenagers in this area have about two folds higher risk of pregnancy compared to teenagers outside the area. The second scanning window for the secondary clusters was found in the Afar region containing 4 clusters which was located at 11.987341N, 40.339030E) with 41.64 km radius, LLR 16.61348, and *p* value < 0.0001. Teenagers inside this cluster have a 5.38 times higher risk of pregnancy as compared to teenagers outside the cluster (Fig. 3).

### Spatial determinates of teenage pregnancy

First Ordinary least squares (OLS) model was fitted for the candidate explanatory variables. In this model, all the requirements of the OLS were met. The Joint Wald Statistic indicated that the overall model significance (*p* < 0.01), the robust probabilities showed coefficient significance (*p* < 0.01) for the explanatory variables. The multicollinearity was also assessed by using the variance inflation factor and there is no issue of redundancy among explanatory variables (VIF < 7.5). The adjusted R^2^ indicated that 19% of the variation in teenage pregnancy was explained by the model. Therefore, the spatial determinates of hot spot areas of teenage pregnancy were being in the poor wealth index, using any type of contraception, using traditional contraceptive methods, and being at secondary educational level (Table 2).

**Table 2:** Summary of ordinary least squares results of teenage pregnancy in Ethiopia, EDHS 2016

Variable	Coefficient	Standard error(SE)	t-statistics	Probability	Robust standard error	Robust t-statistic	Robust probability	VIF
Intercept	0.37	0.052	7.05	< 0.01	0.08	4.34	< 0.01	–
Women from poor wealth index	0.14	0.02	6.06	< 0.01	0.02	5.70	< 0.01	1.40
Contraceptive non users	0.31	0.06	− 5.50	< 0.01	0.09	− 3.32	< 0.01	1.11
Traditional contraception users	0.09	0.03	3.70	< 0.01	0.03	3.11	< 0.01	1.30
Women with secondary education	− 0.09	0.03	− 2.73	< 0.01	0.02	3.38	< 0.01	1.34

OLS diagnosis			
Number of observation	617	Akaike’s Information Criterion (AICc)	− 256.57
Multiple R-squared:	0.19	Adjusted R-square	0.185
Joint F-Statistics	35.97	Prob(> F), (4,612) degrees of freedom	< 0.01
Joint Wald Statistic:	103.20	Prob(> chi-squared), (4) degrees of freedom	< 0.01
Koenker (BP) Statistics:	107.81	Prob(> chi-squared), (4) degrees of freedom	< 0.01
Jarque–Bera Statistics:	334.62	Prob(> chi-squared), (2) degrees of freedom	< 0.01

Although OLS analysis determined predictors of hot spots areas of teenage pregnancy. It assumes that the relationship between each independent variable and teenage pregnancy is stationary across the study area. But this assumption is violated as it is evidenced by significant Koenker (BP) Statistics (*p* < 0.01). This is better handled by the geographically weighted regression (local model when stationary is violated) model. Therefore, a geographically weighted regression model was fitted to produce reliable estimates. In this model, the adjusted R2 value obtained from OLS increased from 0.19 t (Table 2) to 0.30 using GWR (Table 3). This was further supported by a corrected Akaike’s Information Criterion value where GWR provided a smaller (AICc = − 265.62; Table 3) AIC value as compared to a global model (OLS). Since If the AICc values for two models (OLS and GWR) differ by more than 3, the model with the lower AICc is considered to be better [33].

**Table 3:** Geographically weighted regression (GWR) of teenage pregnancy in Ethiopia,2016

Explanatory variables	Women from poor wealth quintiles, non-contraceptive users, traditional contraceptive users, women with secondary education level
Effective number	21.57
Sigma	0.19
Akaike’s Information Criterion (AICc)	− 265.62
Residual squares	22.21
Multiple R-Squared	0.23
Adjusted R-Squared	0.30

**Fig. 4:**
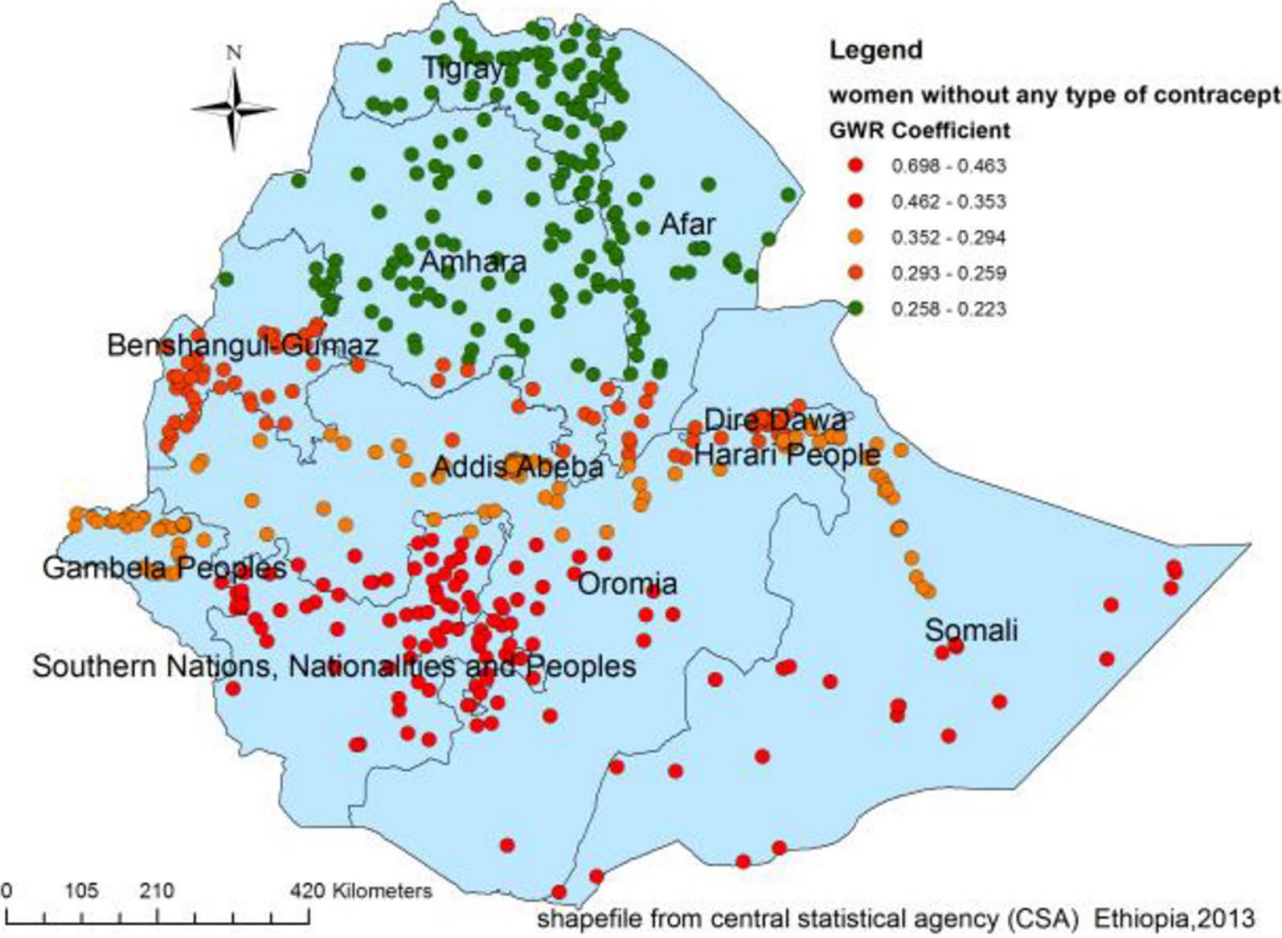
Geographically weighted regression coefficients of contraceptive use to predict teenage pregnancy (source; shapefile from central statistical agency, Ethiopia, 2013)

**Fig. 5:**
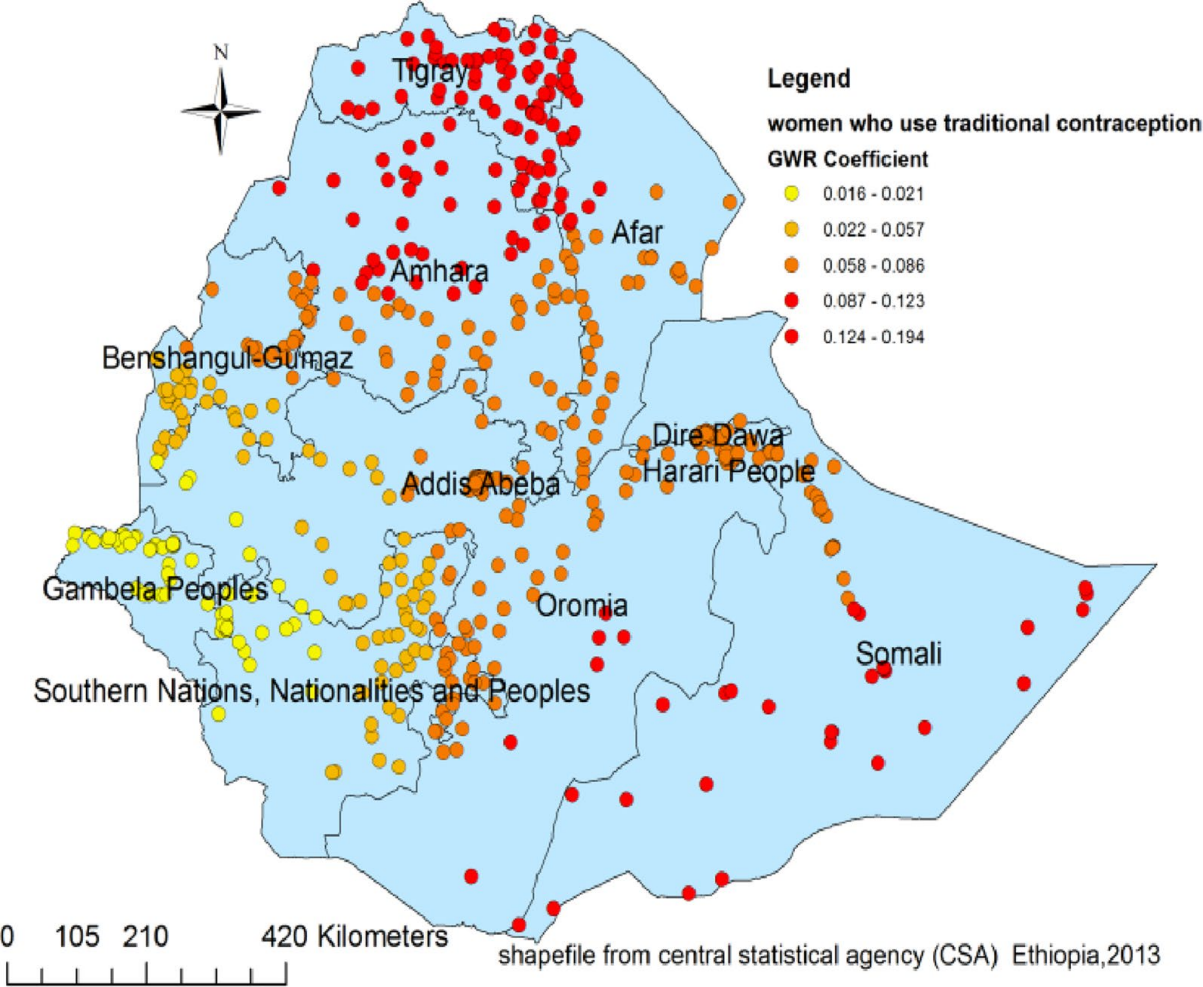
Geographically weighted regression coefficients of traditional contraceptive use to predict the hotspots of teenage pregnancy in Ethiopia (source; shapefile from central statistical agency, Ethiopia, 2013)

**Fig. 6:**
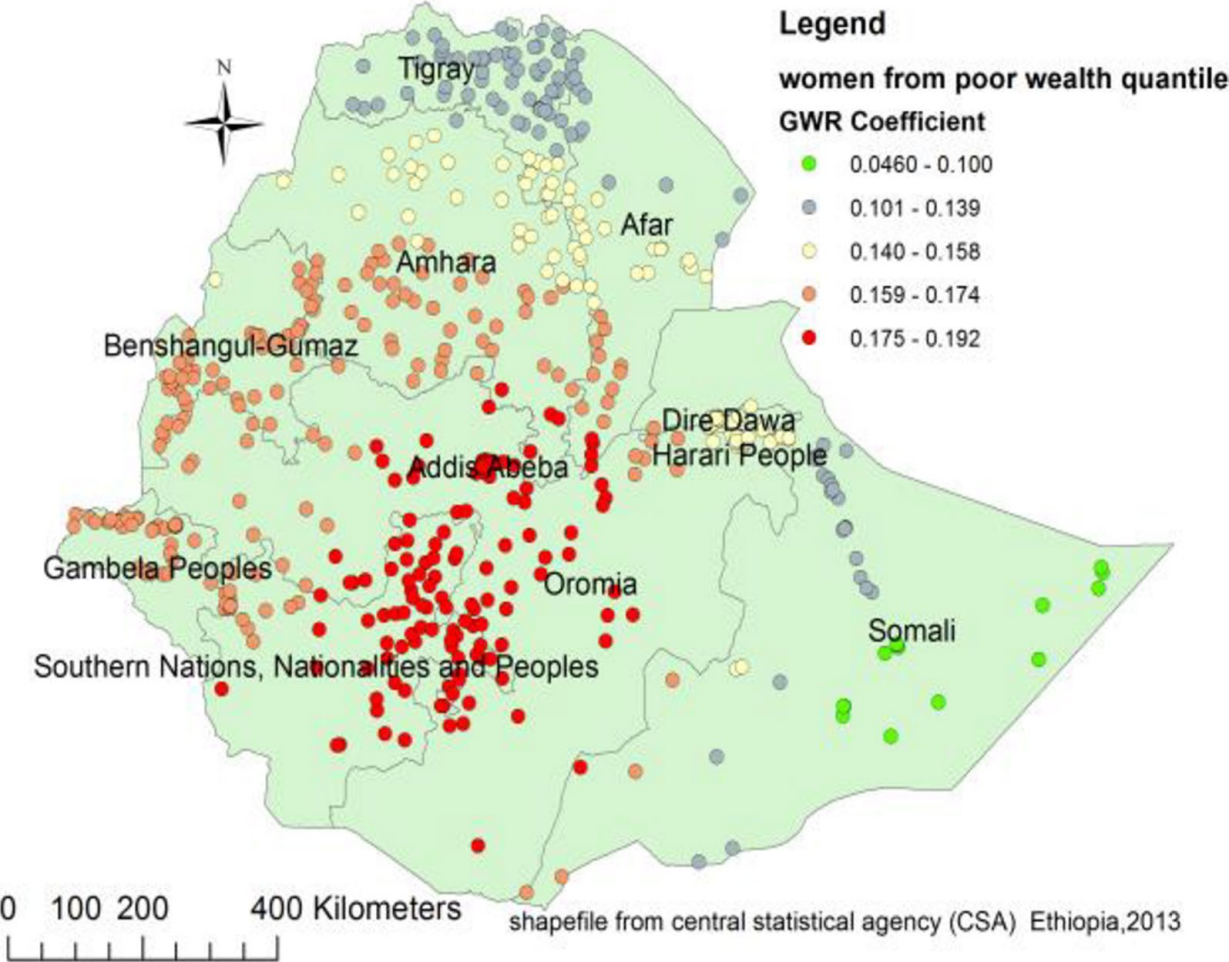
Poor wealth index geographic weighted regression coefficients to predict teenage pregnancy in Ethiopia (source; shapefile from central statistical agency, Ethiopia, 2013)

**Fig. 7:**
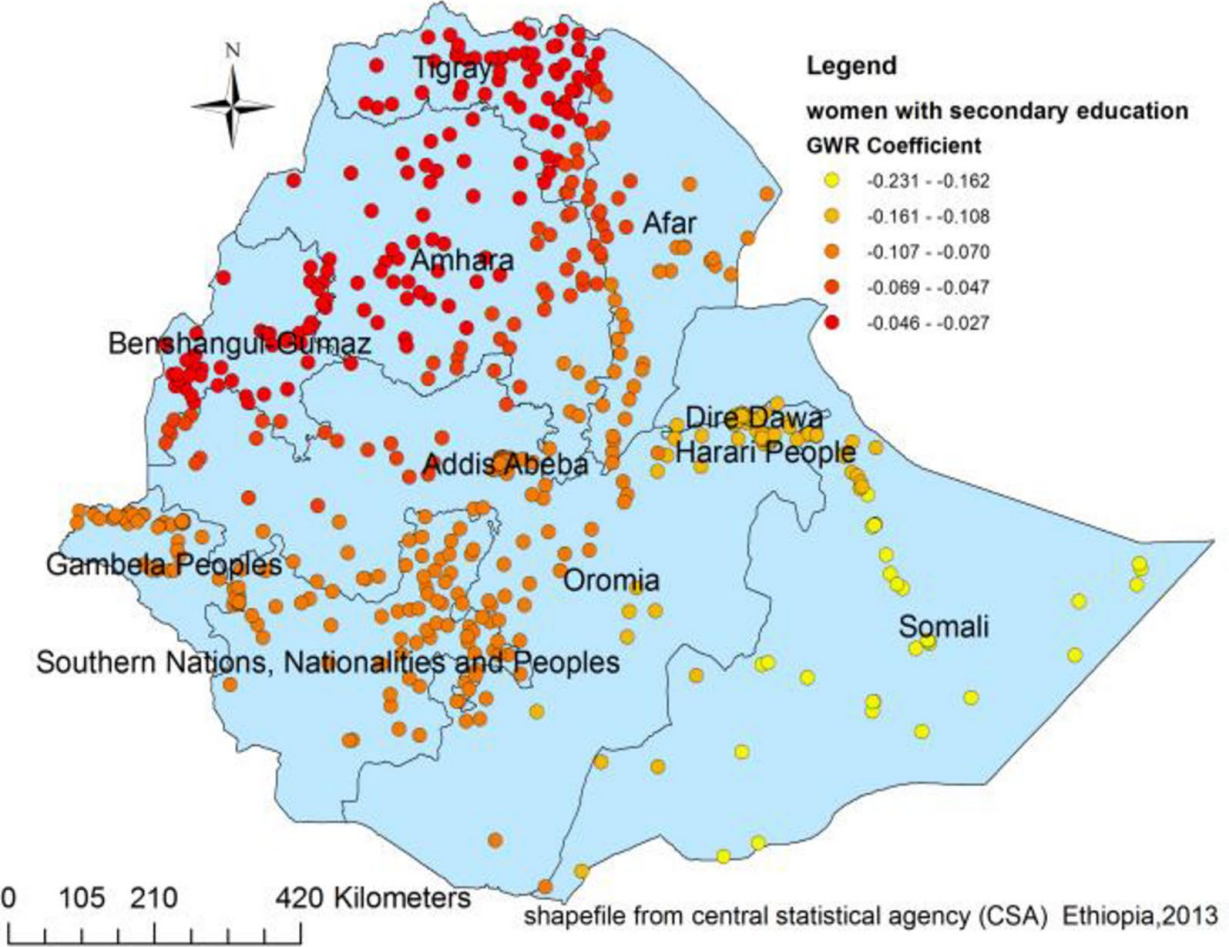
The geographically weighted regression coefficients for women with secondary education to predict the hotspots of teenage pregnancy in Ethiopia (source; shapefile from central statistical agency, Ethiopia, 2013)

Figures 4, 5, 6 and 7 indicate the geographic areas where the independent variables were strong and weak predictors of teenage pregnancy in Ethiopia. For example, not using any type of contraceptive method has a positive relationship with teenage pregnancy. When the proportion of women who did not use any type of contraceptive method increases the occurrence of teenage pregnancy in Somali, south nation nationality and peoples region and Eastern Oromia regions increased. As it was evidenced by larger coefficients in Fig. 4, contraceptive use had a stronger relationship with teenage pregnancy. It was a strong and positive predictor of teenage pregnancy in the southern and southeast parts of Ethiopia.

Similarly, using traditional contraceptive methods was a strong predictor of teenage pregnancy in the Somali, Afar, Amhara, and Tigry regions and Somali Region. On the other hand, the positive and weaker relationship between traditional contraceptive use and teenage pregnancy was observed in Adiss Ababa, Gambela, and the western part of the south nation nationality and peoples region (Fig. 5).

This study also highlights the space dependent relationship between teenage pregnancy and wealth. Consequently, wealth had a positive relationship with teenage pregnancy with the coefficient ranging from 0.046 to 0.192. As the proportion of women coming from households with poor wealth index increases, the incidence of teenage pregnancy also increases in south nation nationality and peoples region, central Oromia, and Benshangul Gumuz regions (Fig. 6).

The other important spatial predictor of teenage pregnancy was women’s education. As a result having secondary education was a negative and strong predictor of teenage pregnancy in Somali, Dire Dawa, and Hareri regions. It depicts that as the proportion of women having secondary education in particular geographic areas increased the prevalence of teenage pregnancy in that specific area decreased. Similarly, secondary education was a moderate and negative predictor of teenage pregnancy in Gambela, SNNP, and in most parts of Afar. However, it has a weak and negative relationship with teenage pregnancy in the northern part of Ethiopia.

## Discussion

This study explored the spatial clustering and spatial predictors of teenage pregnancy in Ethiopia by using different spatial analytic methods. Teenage pregnancy was spatially clustered (Moran’s I = 0.14 and *p* value of < 0.01) in Ethiopia. This finding was consistent with the findings in England [34] and United States [35]. The local level clusters (hot spot areas) of teenage pregnancy were further detected in the Somali, Afar, and eastern parts of Amhara and Hareri regions of Ethiopia. The possible justification could be the cultural variation in age for marriage, the difference in economy, education, media access, and health infrastructures [36–38].

Besides, the teenage pregnancy hot spot areas in the Somali Region and Afar Region might be due to the lifestyle of the community which is characterized by seasonal mobility since the majority of the population live pastoral life [39]. Furthermore, people in these areas have again limited access to health information and services, live in very traditional settings, and adhere strongly to cultural and religious values [40].

The current study also identified the predictors of the hot spots of teenage pregnancy. Not using any type of contraception was a strong positive predictor of teenage pregnancy in Somali, SNNP, and Eastern Oromia regions. Similarly, a positive relationship between using the traditional contraceptive method and teenage pregnancy was observed in the Somali and Afar regions. This might be because of a fear of disapproval by community members due to cultural and religious norms to use contraceptives, which in turn lead to early pregnancy [41, 42]. This is again supported by another literature where women in the eastern part of Ethiopia is either free of any contraceptive or use traditional contraceptive method [39].

This study also highlighted that the proportion of women with a poor wealth index was positively related to teenage pregnancy. The coefficients of this predictor varied from 0.05 to 0.192 with different strengths at different geographic areas. It strongly predicts the occurrence of teenage pregnancy in SNNP, Oromia, Gambela, southern Afar, and Somalia regions. It is expected that women from the poor wealth category might relatively have the limited financial freedom to utilize family planning services. The literature has shown that variation in access to family planning, knowledge of these services, and direct contact with field workers are associated with the wealth gradient [43]. Therefore, a woman with economic challenges might be subjected to unintended pregnancy.

Similarly, education is an important spatial predictor of hot spots of teenage pregnancy. Women with secondary education had a reduced risk of experiencing teen pregnancy in Somali, Dire Dawa, and Hareri regions (Fig. 7). Education might affect the occurrence of teenage pregnancy by influencing women’s health-seeking behavior, primarily the family planning services. Besides, women with higher education are more likely to have a higher level of health awareness, greater knowledge of available health services, improved ability to afford the cost of medical health care, and greater autonomy in making health-related decisions, including choices in family planning [44, 45].

As a strength, the study used data from a nationally representative large dataset, which results in adequate statistical power. Besides, the sampling weight was applied to produce reliable estimates. However, it has the following limitations. First, the aggregated area level findings are generalized to individuals in the area which could lead to ecological fallacy. Second, the location data values were shifted 1–2 kms for urban and 5kms for rural areas for data confidentiality issues. This may affect the exact location of cases.

## Conclusion

Statistically significant hotspots of teenage pregnancy were detected in the Afar, Somali, and Hareri regional states of Ethiopia. geographic areas where a high proportion of women didn’t use any type of contraceptive methods, use traditional contraceptive methods, and a high proportion of women from households with poor wealth quintile had increased risk of experiencing teenage pregnancy. Whereas, those areas with a higher proportion of women with secondary education had a decreased risk of teenage pregnancy.

These findings have valuable policy implications for intervention and program design. They are supremely important for the ministry of health and regional health Bearues to give attention to those hot spot areas in light of those predictors to develop and implement adolescent targeted health programs.

## Data Availability

The datasets we used for this study were publicly available at http://www.dhsprogram.com. Website.

## Abbreviations

CSA: Central statistical agency
EAs: Enumeration areas
EDHS: Ethiopian demographic and health survey
SNNP: South nation nationality and peoples region
OLS: Ordinary least squares
GWR: Geographic weighted regression
